# Improvement of Eruption Disturbance in Mandibular Canine Caused by a Supernumerary Tooth

**DOI:** 10.7759/cureus.70050

**Published:** 2024-09-23

**Authors:** Tatsuya Akitomo, Yuko Iwamoto, Masashi Ogawa, Chieko Mitsuhata, Ryota Nomura

**Affiliations:** 1 Pediatric Dentistry, Hiroshima University, Hiroshima, JPN

**Keywords:** childhood, pediatric preventive dentistry, space maintenance, supernumerary tooth, tooth eruption

## Abstract

A supernumerary tooth is a dental abnormality, which, in some cases, causes disturbance in the eruption of permanent teeth. A seven-year-and-four-month-old female was diagnosed with eruption disturbance in mandibular canine eruption caused by a supernumerary tooth. At the age of eight years and six months, the supernumerary tooth was extracted and space maintenance by a lingual arch was started. Long-term follow-up and interproximal enamel reduction of the primary molar led to the mandibular canine eruption and prevented malalignment. This report highlights the importance of a comprehensive approach to managing supernumerary teeth through timely extraction and space maintenance to facilitate proper permanent tooth eruption.

## Introduction

The oral cavity undergoes major changes during childhood, beginning with the primary tooth eruption through the mixed dentition and ending with the completion of the permanent dentition. Therefore, it is important to continue long-term oral management until the permanent dentition is completed. However, in pediatric dentistry, we may encounter developmental disorders of the teeth, such as abnormal tooth counts and unusual tooth morphology, for unknown reasons [1-4]. A supernumerary tooth is one of these dental anomalies. Surgical treatment is often required to address this anomaly, and long-term follow-up is important to monitor before and after the treatment [2]. The prevalence is 1.2-2.3%, and most of them occurred in the anterior maxilla; however, there were very few cases reported of hypo- or hyperdontia occurring in canine [5,6], and the supernumerary tooth occurring in the canine region is very rare.

Calcified tissue, such as supernumerary tooth or odontoma and dentigerous cyst, may cause adjacent tooth eruption disturbance [2,7,8]. In particular, the presence of an impacted mandibular canine is one of the most difficult challenges that an orthodontist will meet [9]. Therefore, early diagnosis and surgical approach can be important to prevent serious future complications [8].

Herein, we encountered a rare case of supernumerary teeth occurring in a mandibular canine. Although there are many cases in which orthodontic treatment, such as traction, has improved the eruption disturbance, there are few reports of improvement without orthodontic treatment. This report described the progress in improving the eruption disturbance of the canine.

This article was previously presented as a meeting abstract at the 62nd Congress of the Japanese Society of Pediatric Dentistry on May 16, 2024.

## Case presentation

A seven-year-and-four-month-old female came to our hospital with the chief complaint of a supernumerary tooth. She had been receiving oral management from a general practitioner, a radiographic examination at the age of seven years revealed the supernumerary tooth, and she was referred to our hospital. She had no medical or family history.

**Figure 1:**
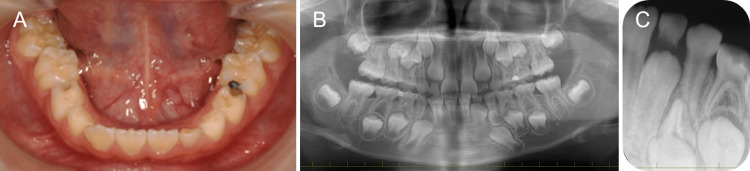
Initial examination at the age of seven years and four months (A) Intraoral photograph; (B) Panoramic radiograph; (C) Periapical radiograph

An intraoral examination showed dental caries in the primary molar and the mandibular left primary lateral incisor was left with light mobility (Figure 1A). Radiographic examination revealed the presence of a supernumerary tooth near the crown of the mandibular left canine, and delayed eruption of the canine was suspected (Figures 1B, 1C). However, the eruption of the left lateral incisor that was not affected by the supernumerary teeth was also delayed compared to the right side. In addition, the mandibular left lateral incisor was in the root formation stage, and the immediate surgical approach had a risk of damaging the root. Therefore, we decided to re-evaluate with a radiographic examination six months later. We referred her to a pediatric dental specialist with dental caries treatment because she lived far away. The mandibular left primary lateral incisor fell out naturally at the age of seven years and nine months. However, we had to extend the interval between dental visits because of the COVID-19 pandemic, and a re-evaluation was performed at the age of eight years and three months.

**Figure 2:**
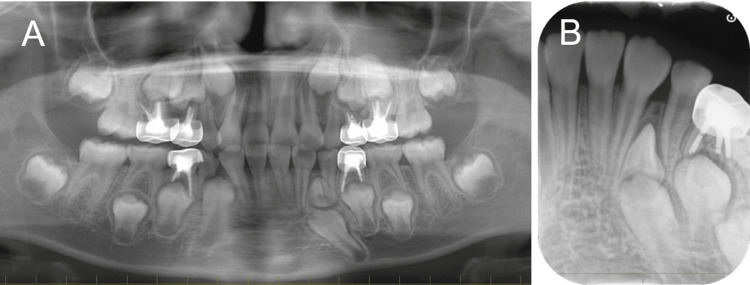
Re-evaluation at the age of eight years and three months (A) Panoramic radiograph; (B) Periapical photograph

Root formation of the mandibular left lateral incisor and delayed eruption of the canine was detected (Figures 2A, 2B).

**Figure 3:**
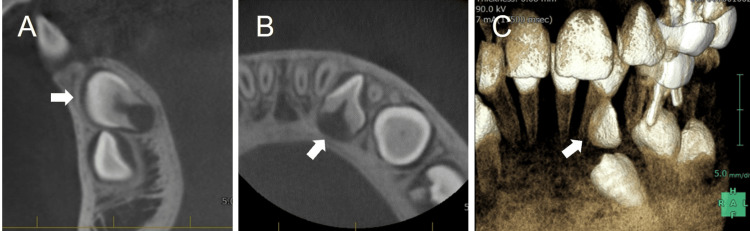
Cone-beam computed tomography images obtained at the age of 8 years 3 months. Arrows indicate the supernumerary tooth. (A) Sagittal section. (B) Horizontal section. (C) Three-dimensional construction image.

The root of the canine was immature. The cone-beam computed tomography images for a more detailed examination revealed the labial inclination of the supernumerary tooth (Figures 3A-3C).

We decided to take a surgical approach, and the extraction of the supernumerary tooth and primary canine was performed in the department of oral surgery under general anesthesia three months later.

**Figure 4:**
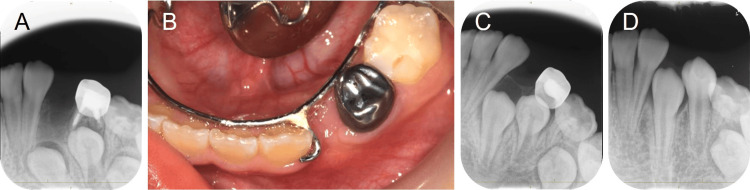
Radiographic examination and intraoral photograph after the extraction of the supernumerary tooth. (A) 8 years and 6 months. (B) 8 years and 7 months. (C) 9 years and 2 months. (D) 9 years and 10 months.

Space maintenance by the lingual arch was started after wound healing (Figures 4A, 4B).

**Figure 5:**
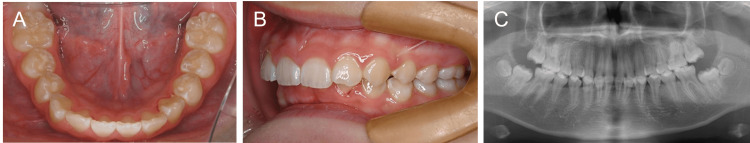
Oral examination at the age of 11 years and 3 months (A, B) Intraoral photograph; (C) Panoramic examination

The direction of the mandibular left canine eruption was gradually improved after the surgical approach (Figures 4C, 4D). The eruption was confirmed at 10 years and 2 months; however, there was insufficient eruption space. Interproximal enamel reduction (IPR), an intentional mesiodistal dimension reduction of the teeth, is a relatively common practice and this is used to gain space in the dental arch for cases of mild-to-moderate crowding [10]. IPR of the mesial surface of the primary second molar at each visit was performed and space maintenance continued until the eruption of the second premolar to utilize the leeway space. Space maintenance was completed at the age of 10 years and 11 months. In addition, intraoral examination and panoramic radiograph at 11 years and 3 months confirmed no pathological findings of the mandibular left canine and no signs of supernumerary teeth (Figures 5A-5C).

## Discussion

We reported on the oral management of the patient with supernumerary teeth occurring in the mandibular canine. Supernumerary teeth may cause delayed or no eruption of adjacent teeth, with a reported prevalence of 24.4% [11]. In addition, Foley (2004) investigated the management of unerupted permanent maxillary incisor teeth following supernumerary tooth extraction and reported that failure of eruption of the permanent maxillary central incisor teeth occurred in 27%, which required surgical exposure or orthodontic treatment [12].

COVID-19 was first detected in China in December 2019 and spread worldwide. The follow-up period for this patient was also postponed due to the COVID-19 pandemic; however, the impact was minimized by early resumption and radiographic examination. The Japanese Society of Pediatric Dentistry has reported the eruption time of the mandibular canine in Japanese females as 10.22 ± 1.03 [13]. The patient was eight years and three months old at the re-evaluation, and the root was immature at that point. In addition, the extraction of the deciduous maxillary canine is one of the interceptive treatment approaches in cases of palatally displaced maxillary canines [14], and primary tooth extraction may contribute improvement of displaced permanent teeth. Therefore, we took a plan to extract the primary canine and supernumerary tooth without traction. As a result, the surgical approach and space maintenance improved the direction of canine eruption, and the natural eruption was confirmed without surgical exposure or orthodontic treatment. This report suggests that only extraction of the supernumerary teeth and primary tooth improves the eruption direction when the affected tooth’s root is immature.

This patient has had dental caries of primary teeth for a long time and there was a mesiodistal space loss, therefore, the stemless steel crown of the maxillary right primary first molar was used for the treatment of mandibular left primary first molar by the pediatric dental specialist. The eruption space of the mandibular left canine was also insufficient at the start time of space maintenance. Although IPR is a part of orthodontic treatment for gaining a modest amount of space in the treatment of crowding [15], we used this method to make an eruption space. In addition, leeway space, the difference between the sum of the mesiodistal crown widths of the primary canines and molars and that of their successors, is clinically crucial in pediatric dentistry [16,17]. Vyas et al. (2016) reported that preserving leeway by the lingual arch has the potential to eliminate incisor crowding [18]. In the present case, although the mandibular left canine erupted at 10 years and 2 months, we continued the space management until the age of 10 years and 11 months, the completion of the mandibular second premolar eruption. Preserving leeway, and eruption guidance by IPR prevent the crowding.

When the spontaneous eruption of the mandibular canine is deemed to be unrealistic, the treatment plan includes no treatment, surgical exposure and orthodontic traction, the surgical extraction of the impacted canine followed by either orthodontic space closure or future prosthetic replacement or transalveolar relocation [19]. Abouei Mehrizi et al. (2010) reported a patient with bilateral mandibular supernumerary occurring in the canine region and both orthodontic treatment and supernumerary tooth extraction were needed [20]. Zhang et al. (2024) reported the management of a horizontally impacted permanent mandibular canine, including orthodontic treatment, three-dimensional radiology, biomodel fabrication, and subsequent endodontic treatment [19]. Early diagnosis may be an interceptive opportunity to encourage the spontaneous eruption of mandibular canines by primary mandibular canine extraction [19], which reduces the burden on the patient. We reconfirm the importance of early diagnosis and an appropriate treatment plan for a supernumerary tooth.

## Conclusions

We encountered a rare case of eruption disturbance of the mandibular canine caused by a supernumerary tooth. Some cases require not only extraction of the supernumerary tooth but also orthodontic treatment such as traction. The extraction of the supernumerary tooth at a time when the root of the affected tooth was immature led to the tooth's natural eruption without orthodontic treatment. In addition, proper space management prevented the malalignment. We highlight the importance of appropriate diagnosis, treatment, and long-term oral management of eruption disturbance caused by a supernumerary tooth.
